# Does Animal Personality Affect Movement in Habitat Corridors? Experiments with Common Voles (*Microtus arvalis*) Using Different Corridor Widths

**DOI:** 10.3390/ani9060291

**Published:** 2019-05-29

**Authors:** Gabriele Joanna Kowalski, Volker Grimm, Antje Herde, Anja Guenther, Jana A. Eccard

**Affiliations:** 1 Animal Ecology, Institute of Biochemistry and Biology, University of Potsdam, 14469 Potsdam, Germany; eccard@uni-potsdam.de; 2 Ecological Research Station Gülpe, University of Potsdam, 14715 Havelaue, Germany; 3 Department of Ecological Modelling, Helmholtz Centre for Environmental Research-UFZ, 04318 Leipzig, Germany; volker.grimm@ufz.de; 4 Plant Ecology and Nature Conservation, University of Potsdam, Am Mühlenberg 3, 14476 Potsdam, Germany; 5 Department of Animal Behaviour, Bielefeld University, 33615 Bielefeld, Germany; antje.herde@gmx.de; 6 Department of Evolutionary Biology, Bielefeld University, 33615 Bielefeld, Germany; guenther@evolbio.mpg.de; 7 Department of Evolutionary Genetics, Max Planck Institute for Evolutionary Biology, 24306 Plön, Germany

**Keywords:** activity, animal personality, wildlife corridors, habitat connectivity, individual differences, rodents

## Abstract

**Simple Summary:**

An animal’s personality may affect how they move and what risks they take while they are moving within a landscape. Understanding the movement constraints of wildlife is of increasing importance in fragmented landscapes. We investigated how rodents of opposing personality types moved through two experimental corridors of differing widths. We tracked the voles with automated radio telemetry and quantified the effects of personality on movement. While personality measures did not predict movement patterns, voles in the narrow corridor system entered the corridor faster and spent less time in the corridor than those in the wide corridor. Thus, it may be impossible to detect differences in the risk-taking behavior of small rodents based on personality types if their perceived predation risk is too high. Regarding corridors, our results suggest that the widely held principle that wider is better may not hold true if the fast exchange between populations individuals is the designated function of the corridor.

**Abstract:**

Animal personality may affect an animal’s mobility in a given landscape, influencing its propensity to take risks in an unknown environment. We investigated the mobility of translocated common voles in two corridor systems 60 m in length and differing in width (1 m and 3 m). Voles were behaviorally phenotyped in repeated open field and barrier tests. Observed behavioral traits were highly repeatable and described by a continuous personality score. Subsequently, animals were tracked via an automated very high frequency (VHF) telemetry radio tracking system to monitor their movement patterns in the corridor system. Although personality did not explain movement patterns, corridor width determined the amount of time spent in the habitat corridor. Voles in the narrow corridor system entered the corridor faster and spent less time in the corridor than animals in the wide corridor. Thus, landscape features seem to affect movement patterns more strongly than personality. Meanwhile, site characteristics, such as corridor width, could prove to be highly important when designing corridors for conservation, with narrow corridors facilitating faster movement through landscapes than wider corridors.

## 1. Introduction

Behavioral traits differ consistently between individuals of the same species or population over time and across situations and are often highly repeatable within the same individual. Individuals may, for example, vary in their propensity to investigate unknown objects or areas, commonly called boldness and exploration, respectively. Usually, these differences between individuals fall along a continuum in which the extreme values are often referred to as ‘bold’ or ‘shy’ and ‘non-explorative’ or ‘explorative’. These traits, which are referred to as animal personality [1,2,3], can influence various aspects of movement in space and time, e.g., dispersal [4,5]. As with personality, how wild animals use space also varies consistently between individuals, which could be attributed to personality type [6,7]. Asocial mosquito fish (*Gambusia affinis*), for example, have been found to be more likely to disperse and to travel longer distances than their social conspecifics [8]. Likewise, exploration activity measured in a standardized test setup influenced space use under natural conditions in rodents [9] and birds [10,11], as well as habitat choice in rodents [12].

Personality traits, such as exploration tendency, can influence how individuals perceive and react to varying habitats. For instance, deciding whether to hide, be active, or remain in or disperse from a habitat may depend on personality aspects. Thus, certain habitat types may only support the movement for a fraction of the population. Habitat corridors are critical for connecting populations via the exchange of individuals and genetic information in fragmented landscapes [13,14,15,16]. Functional connectivity may thus be determined not only by the characteristics of the habitat corridor [17], but also by the individual characteristics of the moving animals using it [18]. Habitat features such as width [17,19], length [20,21], geometric shape [17], vegetation structure (e.g., within hedgerows [22,23,24], grass strips [25,26], or riparian buffer strips [27,28]), and the surrounding matrix [29] often affect functional connectivity for woodland plants, invertebrates, and vertebrates. Studies on large mammals using GPS-tracking or similar techniques mainly highlight corridor width and continuity, or length, as being crucial for connectivity [30,31,32,33]. Furthermore, while the exact dimensions depend on the focal species’ size and needs, generally speaking, it is the width of efficient corridors that enhances the probability and frequency of crossings among mammalian predators [27,30,31,32], rodents [34], birds [20], and large African mammals [33]. Thus, conservationists frequently advocate a general principle that wider is better when it comes to corridors [19,35].

Previous studies on voles comparing corridor widths > 10 m found no effects of width on movement (e.g., References [36,37]). However, studies using corridors 1 m and 3 m in width indicate differences in use (e.g., References [35,38]). Andreassen et al. [35] show that widths of 1 m allow higher connectivity than 3-m-wide corridors. Meanwhile, Lorenz and Barret [38] show that vegetation cover supplemented by a fence increased dispersal in 1-m-wide corridors. Based on these findings, we replicated the methods for testing corridor use and connectivity, but adding the personality component to the experimental setup and using a natural setting without fences.

In this study, we assumed that a narrow corridor poses a larger predation risk for prey animals, since it increases the chance of encountering predators, which often forage along with ecotones and landscapes structures [39]. Thus, we investigated the effects of animal personality on movement patterns in corridors, using small mammals in experimental grassland corridors as a study system. Considering that small mammals experience high aerial predation pressure and thus feel safer in tall grass [40,41,42,43], we used the overall design of earlier studies where grass strips of differing width or length and with high vegetation are used as habitat for small mammals, while the matrix surrounding those structures consists of low vegetation (e.g., References [35,36,37,38,44,45,46]). 

The common vole (*Microtus arvalis*) is a ground-dwelling species inhabiting grassland and fields all over Europe [47] and feeds on forbs and grasses [48]. Adults weigh between 25 to 40 g (females) or up to 50 g (males) [48]. The breeding season starts in April and ceases in fall with annual density fluctuations [43,48]. Common voles live in dense colonies and experience high predation pressure [49].

We predicted that animals with higher degrees of exploratory behavior in standardized personality tests would be more prone to explore and enter the corridor when released to an unknown environment than animals with lower exploration scores in standardized tests. We additionally expected interaction between animal personality and corridor width. Specifically, we expected more explorative animals to be more mobile than less explorative animals in the narrow corridor than in the wide corridor, which presented them with less space to hide from potential predators. Alternatively, as animals become more sensitive to risk while dispersing through unknown areas, sensitivity to risk may also be elevated after a forced translocation and site characteristics such as corridor width may explain movement better than the animal’s personality. Along with the analysis of personality impact on corridor use, we investigated its effect on daily activity patterns, as suggested by other studies [50,51,52]. Distribution of activity phases did not differ between night and morning, implying that neither the personality nor the corridor widths affected the polyphasic activity patterns of common voles.

## 2. Materials and Methods 

### 2.1. Study Area and Animals 

The study was conducted between July and October 2017close to the ecological research station of the University of Potsdam (52°44′00.0″ N 12°12′41.7″ E) in Gülpe, Germany. The station is surrounded by mesic grassland which is mowed twice a year.

We captured common voles (Microtus arvalis) using Ugglan-type traps (special No2, Grahnab, Sweden) at five trapping sites (N = 7–24 traps per site) near the corridor systems (40–100 m). Traps were baited with rolled oats and apple and modified with an escape opening for shrews [53]. Due to high daytime temperatures and lack of shade on the grassland, we set traps mostly at night for animal welfare reasons. On cooler days, traps were also activated during the day. Traps were activated for a maximum of 12 h. To carry very high frequency (VHF) radio transmitters, male voles with a weight of > 22 g were selected, as radio transmitters should weigh < 5% of an animal’s weight [54]. We did not capture and translocate females, as they almost constantly nurse nestlings at unknown nest sites during the mating season. The selected males were behaviorally tested 2 h (median, 10 min to 6 h) after capture. Thereafter, males were transferred to a cage for housing.

Voles were housed at the research station in a room with a window and kept singly in standard polycarbonate cages (Ehret GmbH Germany, Typ III, Mahlberg, Germany—42 cm × 27 cm × 16 cm) containing wood shavings, hay, fresh grass, and paper rolls for shelter. Water and food pellets (Ssniff V1594 R/M-H Ered II) were available ad libitum and slices of carrots and cucumber were added daily. The bedding was changed once a week. The voles were marked permanently with a passive integrated transponder (‘PIT’; Trovan ID-100; 2.12 mm × 11.5 mm, 0.1 g).

After 8−14 days, we repeated the tests. Depending on the suitability of weather conditions for VHF telemetry, voles were collared and transferred 10−27 days after capture into one of the corridor systems for movement observations, which lasted 48 h. After recapture, the VHF transmitter was removed and behavior was sampled a third time, i.e., 12−43 days after the first behavioral test (Supplementary Materials Figure S1). After the experiment we released the voles at a distance of >250 m from the experimental plot to prevent them from returning.

### 2.2. Personality Tests

Fifty-six voles were phenotyped for behavior. Each behavioral testing round consisted of a barrier test and an open field test [55]. Each part lasted for five minutes and was observed directly by the same observer. To avoid touching and handling the voles, we released them individually from their trap into a plastic box and transported each vole between box or cage and arenas in its own transport pipe (PVC, diameter 5 cm, length 11 cm).

#### 2.2.1. Barrier Test

We conducted the barrier test in a semi-transparent plastic box (45 cm × 22 cm × 25 cm) covered by a plastic lid. A 4.5-cm-high gray plastic barrier divided the box into two equal compartments. Variables measured were: Latency to cross the barrier (if no crossing occurred, time was set to 300 s), the activity of the vole every 10 s with instantaneous 1-0-sampling (‘1’ describes any kind of movement or grooming, while ‘0’ illustrates no movement or sitting up and looking around), and ‘crossing frequency’ (crossings per min during time interval left after subtraction of latency).

#### 2.2.2. Open Field Test

The open field test was conducted in a round, metal arena (1-m diameter, 35-cm-high wall). We defined the wall zone as safe (the width was related to vole length, i.e., approximately 10 cm) and the center as unsafe [56]. We released the voles in the center of the arena and measured the latency to reach the wall. We also collected data on the latency to re-enter the unsafe zone of the arena, the activity status of the voles every 10 s, and the number of rests in the unsafe zone (max. 30 samples) with instantaneous 1-0-sampling every 10 s. If a vole did not re-enter the unsafe zone, the latency was set to 300 s.

### 2.3. Corridor Test

#### 2.3.1. Setup of Experimental Plot

**Figure 1:**
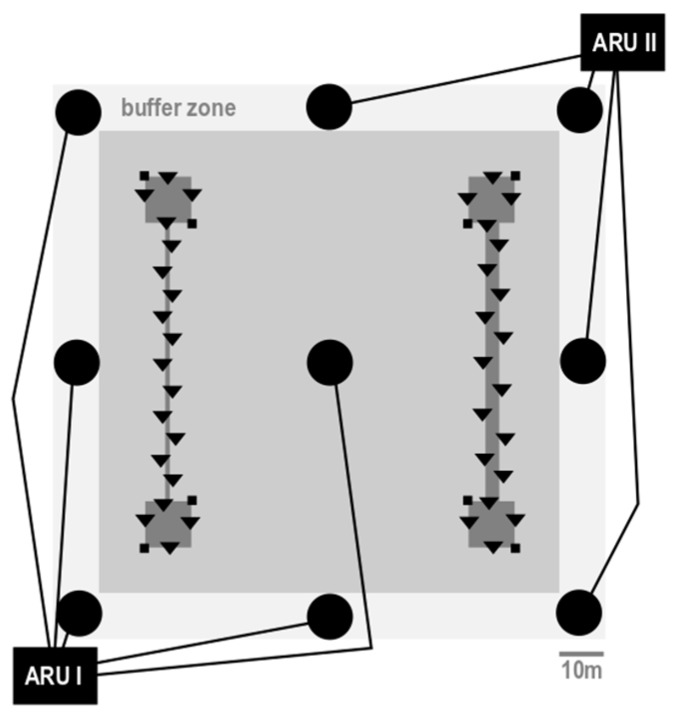
Configuration of corridor systems in grassland with corridors and patches. Black boxes = ARUs (automated receiving units for telemetry); black circles = omnidirectional antennas; black lines = antenna cables. Black triangles = stationary traps; small black squares = calibration transmitters; dark grey squares and rectangles = corridor systems; light grey = matrix area within the experimental site; very light grey buffer zone = matrix outside the experimental site. Corridor systems were 70 m apart and separated by a mown matrix, corridors were 60 m long.

The unfenced experimental plot covered an area of 120 m × 130 m. Vegetation was mowed except for two experimental corridor systems (Figure 1). Each corridor system consisted of two 10 m × 10 m habitat patches (vegetation height 34 ± 8 cm) connected by a 60-m-long grass corridor (height 42 ± 15 cm). The habitat patches potentially provided enough resources for a vole to stay if they are reluctant to leave, since reported home ranges (125 m^2^ (minimum convex polygon with 30 m^2^ 95% Kernel) [57] to 202 m^2^ (SE = 54 m^2^) [41]) also include overlaps of several individuals and daily movement distances as short as 9−49 m (median = 20 m) [57]). Thus, males could choose to stay within the patch or to explore and leave. On the other hand, the scale of the systems was explorable for a common vole, with reported dispersal distances between 8 and 457 m, while most (64%) travel less than 100 m [58], but have the ability to cover distances of 500−1500 m overnight [48].

The systems differed in the width of the corridor (1 m and 3 m). Vegetation did not differ among corridor systems, with a mean vegetation height of 37 ± 9 cm (t = 0.43, *p* = 0.66, N = 26 measurements taken every 10 m) and the same proportion of locations with 8 measures of ‘dense’ and 18 of ‘very dense’ vegetation in each of both corridors. Corridor systems were surrounded by a matrix of mown vegetation with a distance of 70 m between systems and >20 m to the edges of the experimental plot (comprised of the matrix area of 10 m and an additional buffer zone of 10 m outside the experimental plot) (Figure 1). The matrix and the buffer zone were kept short (height < 10 cm) by mowing every 3–4 weeks and the edges of each corridor system were trimmed every week (height 6 ± 0 cm) to discourage voles from leaving the corridor system. Only one individual left its corridor system and entered the other, hence corridor systems did not affect each other.

We reduced and monitored the resident vole population in the corridors by live-trapping with 19 stationary traps (Figure 1) before each experimental run. We removed 107 animals in total from both systems during 25 trapping events, with 57 from the narrow system and 50 from the wide system. We did not remove adult, lactating, resident females from either of the corridors to avoid separating them from their nestlings at unknown nest sites. Thus, both corridors contained 3-5 females throughout the study period. Since roaming animals will always meet residents and encounter the scent of other animals in nature, we considered both to be a vital part of the corridor systems in a natural setup. If suitable in size and age, males trapped from the systems (N = 15) were used for the experiments, but released to whichever corridor system was unknown to that individual.

#### 2.3.2. Movement in the Corridor System

We released 34 to the corridor systems in nine 48-h experimental runs. Each experimental run was conducted with four males—two for each corridor system. The individuals in each pair released differed as much as possible in their personality, but as little as possible in weight to avoid larger males dominating smaller males [59]. We used the means of two variables from both the barrier and the open field tests to generate two ranking values. The first rank was based on the number of crossings and the activity measure from the barrier test, while the second rank was derived from the latencies to cross the barrier and to enter the unsafe zone from the open field test. The ranking resulted in a gradient for each subgroup from which we chose males of opposing personality ranks, A total of 16 voles were released into the narrow corridor (Supplementary Materials Figure S2J–Q) and 18 into the wide corridor (Supplementary Materials Figure S2A–I) (mean weight = 27.5 g). 22 males which were used in the behavior tests were not chosen for release in the corridor system as they lost weight while being housed and were no longer heavy enough to wear radio collars. The behavioral data obtained from these individuals was nevertheless used in the determination of repeatability of behaviors, since this allowed for the calculation of repeatability with more precision [60].

Males were equipped with a VHF radio transmitter (BD-2C, Holohil Systems Ltd., Canada, 1.1 g) on collars between 07:00–10:40, approximately. Two voles with opposing personality types were released simultaneously to the north patch of their respective corridor system in the late morning before a midday break in vole activity (1020 to 1324 h). In two cases, we had to start the movement observation although one vole from the previous experiment, which we had not yet recaptured, was still in the corridor system. After 48 h, tracking was terminated and we started removing the voles from the corridor systems. We restricted our analysis to voles that spent the full 24 or 48 h tracking period in the systems (N_Day 1_ = 22, N_Day 2_ = 21). Some voles could not be observed for the full tracking period within the corridor systems (Supplementary Figure S2B,C,E,H–J,N,P,Q); 11 left the systems before tracking was terminated (Supplementary Figure S2B,C,E,H–J,N,P,Q), henceforth referred to as ‘escapees’, and a twelfth vole was lost to predation after the first 40 min of the tracking period. 36% of escapees left during daylight and neither the distance of the trapping site to the corridor systems (trapping sites < 100 m and > 100 m: Chi^2^ = 0.74, *p* = 0.39) nor the corridor system itself (Chi^2^ < 0.001, *p* = 1) influenced the likeliness that a vole would escape the grid. In one run, we had technical problems with the tracking system, thus data collection stopped after 1820 h (Supplementary Materials Figure S2E,N).

**Figure 2:**
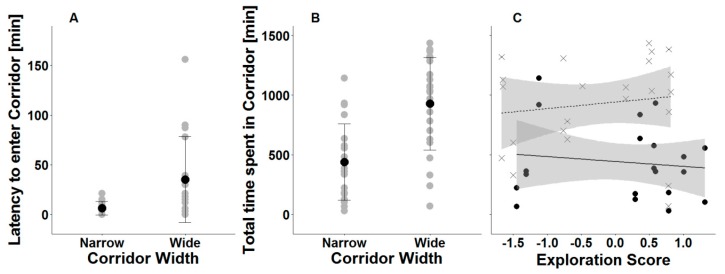
Movement in the corridor system. (**A**) Original values, means, and standard error of latencies of N = 33 to enter the corridor for the first time depending on corridor width. (**B**) and (**C**) The total time N_Day1_ = 22 and N_Day2_ = 21 male common voles spent in a grassland corridor within 24 h. (**B**) Original values, means, and standard error. (**C**) Modelled values from mixed effects model, including the terms month and experimental day (two days per individual) as covariates, with ID and ID of the second male in pair as random factors. Dots represent animals in the narrow corridor and crosses represent animals in the wide corridor. Each animal is represented by two symbols (same exploration score, but different days). The solid line represents the regression slope for the narrow corridor and the dashed line represents the regression slope for the wide corridor. The grey bands display the confidence interval of the model.

The movement was observed using an automated VHF-telemetry tracking system [12,61]. The system consisted of two automated, multi-channel receiver units (ARU, JDJC Corp./Sparrow Systems, Fisher, IL, USA), eight omnidirectional antennas (Winkler-Spezialantennen, Groundplaneantenne GP 150, 150 MHz, http://www.winklerantennenbau.de/verti.htm) mounted on 1.7-m-high poles surrounding the experimental plot, and an additional antenna in the center (Figure 2). The ARUs were programmed to first record five to nine beacons (VHF radio transmitters at known positions for calibration) once, followed by the vole transmitters, which were recorded seven times per 3-min recording cycle (with one exception when the transmitters were recorded every 4 min for the first 7.5 h). Median signal strengths of the seven recordings were used for calculated positions. This process resulted in one position being recorded every 3 min per vole and a total of 960 automatically-collected locations per animal.

We calibrated the tracking system with 179 known positions. The corridor width was smaller than the error of the system (error considering both axes—7.04 ± 8.31 m). Therefore, we only analyzed the signal position along the axis parallel to the corridor to analyze movement between patches and within the corridor, similarly applied by Briner et al. [62] when indicating home range size by home range length in a weed strip. Frequent monitoring via handheld telemetry (Yagi-Antennae of TVP Positioning AB (Type No: A11-0200) and receiver of Telonics (Model TR-5)) was performed to confirm that the voles were still in the corridor (8−12 positions per animal during daytime). The linear position was predicted from the distribution of logged signal strengths by means of linear regressions (R^2^ = 0.84−0.91 with known positions in the different replicates). We smoothed the calculated locations using the median of three subsequent positions (over a total of 9 min) to reduce the effects of changes in posture (which also creates variation in signal strength) relative to changes in location. To correct for the distortion of the calculated positions relative to the real positions, we placed beacons at the entrance of the corridor (error 4.6 ± 6.6 m) while the radio tracking was taking place (Supplementary Materials Figure S2).

Analyzing the position changes over time (Supplementary Materials Figure S2), we generated the following variables. The first set includes variables describing individual differences in the tendency to explore a new environment: Latency to enter the corridor for the first time and latency to arrive at the south patch for the first time (describing an individual’s propensity for small versus large exploration bouts). The second set of variables incorporates connectivity: Latency to return to the start patch after visiting the south patch, number of completed trips between both patches, and number of changes in direction while travelling through the corridor (directness of movement, with fewer changes in direction, indicating higher connectivity). The third set of variables describes perceived risk of the environment: Number of visits to the corridor and total time spent inside the corridor (describing an individual’s propensity to familiarize itself with the new area), number of visits to the north and south patches of the corridor, and total time spent in either patches. However, the total time spent in each patch or corridor may report similar information like the number of visits to each patch and corridor.

Except for the latencies (‘latency to enter the corridor for the first time’, ‘latency to arrive at the south patch for the first time’, ‘latency to return to the start patch after visiting the south patch’), we created all variables for the first and second 24 h separately.

### 2.4. Statistical Analyses

#### 2.4.1. Personality

In total, 56 voles were tested in 152 behavioral tests. The number of repeats per behavioral test varied among individuals, due to their use in the experiment or missing recaptures, i.e., 1 vole was tested once, 15 voles were tested twice, 39 voles were tested three times, and 1 vole was tested four times.

All personality and movement data were analyzed using the free software R (version 3.5.1) [63]. We used the full personality test data to calculate the repeatability scores of behavioral test variables and calculated a general personality score for further analysis of the movement variables. For this reason, the variable ‘latency to re-enter the unsafe zone of the arena’ was transformed into a binomial variable (entering unsafe zone/not entering unsafe zone), since voles did not re-enter in 40 tests during the testing period. Potential ecological and individual-specific influences on each variable were explored by including several fixed effects, as well as animal ID as a random factor in univariate mixed models. Body mass; trapping location; season; test interval (time since the last behavioral test); time of day; light condition during the experiment; test round; the starting site for the barrier test; and, for the open field test, the time between the barrier test and open field test were initially included as fixed factors. Non-significant effects were removed from the model step-wise. Residuals of the models were inspected visually for homogeneity of variances and Gaussian distribution using qq-plots. Models were built using the lme4-package [64] and p-values were derived using the lmerTest package [65]. We calculated raw repeatability using an intercept-only model and conditional repeatability by including fixed effects that significantly affected behavioral performance, following Nakagawa and Schielzeth [60]. Repeatability calculations were conducted using the rptR package [66]. We used those behavioral variables which showed significant raw repeatability to perform a principal component analysis (PCA). To fit the assumption of independence of data points in a PCA, we calculated the means of the behavioral scores across all test repeats and used one value for each vole to run the PCA. We used the PCA to reduce the number of variables describing behavioral tendencies and tested our hypotheses following a mixed model framework. Principal components (PC) with eigenvalues above 1 were kept for further analysis (Kaiser-Guttman criterion; [67]).

#### 2.4.2. Movement in the Corridor System

To select a set of independent variables that describe different aspects of movement patterns, we first decided on biologically valuable variables and removed any redundant variables which were intrinsically represented by others (based on high Spearman correlations and investigated from the movement variables (rho > 0.3), Supplementary Materials Table S3). Correlations were estimated with the function ‘rcorr’ (package Hmisc).

We further analyzed the non-correlated movement variables ‘latency to enter the corridor for the first time [min]’, ‘latency to return to the start patch after visiting the south patch [min]’, ‘total time spent in the corridor [min]’, ‘total time spent in the south patch’ [min]. Three of these variables except ‘latency to return to the start patch after visiting the south patch [min]’ may indicate the explorative behavior and the willingness of a vole to use the corridor, while the latency may describe the connectivity of the corridor. Furthermore, the total time spent in the corridor correlated strongly with the number of changes in direction while travelling through the corridor (rho = 0.98, *p* < 0.001), and the latency to enter the corridor correlated with the total time spent in the north patch (rho = 0.4, *p* = 0.02) (Supplementary Materials Table S3). Additionally, we compared the personality measure of voles which did not leave the system during the experiment with that of escapees by using the Mann-Whitney-U-test.

Depending on the variable’s distributions, we used LMMs and GLMMs (lme4-package, [64]) to investigate whether personality score or corridor width explained the variation in the observed movement variables. Residuals of the models were visually inspected for Gaussian distribution by using qq-plots and homogeneity of variances to test model fit. P-values were obtained using the lmerTest package [65].

The latency to enter the corridor was zero-inflated, due to the measurement method. Therefore, we used a two-part model, the ‘zero-altered negative binomial’ (ZANB) model. The ZANB model was compiled with a hurdle count model using the pscl-package [68]. The total time spent in the south patch [min] was adequately modelled by a GLMM with an assumed Poisson distribution.

For the four movement response variables, we ran separate models, including the calculated PC score and the corridor width as explanatory variables. Furthermore, we analyzed the influence of the month when testing occurred as a fixed factor and the paired male within the same corridor system as a random factor in all models. In two models, excluding both latency models, the day of the observation (first or second) was included as a fixed factor and the animal ID as a random factor. We report means and standard errors unless stated otherwise (see also Supplementary Materials Table S2). We compared the influence of random factors by calculating the marginal and conditional R^2^ [60].

### 2.5. Ethical Note

The study followed all applicable international guidelines, German national laws, and protocols from the ethical commissioner for animal experiments at the University of Potsdam. Experiments were part of a project under the permission of the Brandenburg State Office for Occupational Safety, Consumer Protection and Public Health (Landesamt für Arbeitsschutz, Verbraucherschutz und Gesundheit [LAVG]) (reference number 2347-32-2017). Voles were captured with permission from the State Office of Environment, Public Health, and Consumer Protection (Landesamt für Umwelt, Gesundheit und Verbraucherschutz [LUGV]), now known as the State Office of Environment (Landesamt für Umwelt [LfU]) (reference number LUGV_RW7-4744/41+5#243052/2015, AZ. N 1 0424). Housing was permitted by § 11 Nr. 1a of the German Animal Protection Act (reference number: 386-1-).

## 3. Results

### 3.1. Personality

Of the seven behavioral variables scored by means of the barrier and open field tests, four were highly repeatable (crossing frequency R = 0.35, activity in the barrier test R = 0.31, activity during the open field test R = 0.39, entering the unsafe zone in the open field R = 0.24, *p* < 0.05, N = 56), (Supplementary Materials Table S1).

All behavioral variables were highly correlated with each other, ranging from rho = 0.5 to 0.8, *p* < 0.001. Hence, a PCA resulted in one main component, which explained 72% of the variance. The behavior variables ‘crossing frequency’, ‘activity in the barrier test’, ‘entering unsafe zone’, and ‘activity during the open field test’ loaded onto the main component with 0.83, 0.90, 0.84, and 0.82, respectively (N = 34).

The principal component reflects the explorative behavior of common voles, with increasing values representing a higher degree of exploration and activity, similar to the results found in Herde and Eccard [55]. Hereafter, we will refer to the PC-score as exploration score. The score ranged from −1.87 (not explorative) to 1.56 (very explorative) (N = 33).

### 3.2. Movement in the Corridor System

Here, we present results of uncorrelated behavioral variables (rho < 0.3) to investigate if personality affects independent patterns of movement. However, a complete analysis of all movement variables (Supplementary Materials Table S4), as well as a correlation matrix (Supplementary Materials Table S3) can be found in the supplementary material. For all variables, the results are in accordance with the ones presented here.

The latency to enter the corridor ranged from 0 to 156 min (21 ± 34 min, N = 33) and voles spent between 27 to 1434 min (700 ± 431 min, N_Day 1_ = 22, N_Day 2_ = 21) within the corridor each day. The latency to return to the start patch after visiting the south patch ranged from 105 to 1992 min (802 ± 685 min, N = 14) and voles spent between 0 to 1407 min (476 ± 518 min, N_Day 1_ = 22, N_Day 2_ = 21) in the south patch each day. Animals which were tested in September spent less time in the south patch (356 ± 424 min, t = −2.2, *p* = 0.027, N_Day 1_ = 15, N_Day 2_ = 14) than those tested in August (726 ± 616 min, N_Day 1_ = 14, N_Day 2_ = 14) and they tended to spend more time in the corridor (810 ± 383 min, t = 1.9, *p* = 0.07) than their counterparts tested in August (471 ± 448 min). On the first day of testing, the voles spent more time in the south patch at the opposite end of the corridor from where they were released (488 ± 525 min, t = 6.6, *p* < 0.001, N = 22) than they did on the second day (464 ± 523 min, N = 21) (Table 1). Descriptive statistics across all movement and activity variables are given in Table S2 of the supplementary material.

Males that stayed in the corridor system for the full 48 h did not differ in their exploration score (Score: −0.2 ± 1.1, N = 22) from escapees (Score: 0.4 ± 0.8, N = 11, Mann-Whitney-U-test: W = 152, *p*‑value = 0.2). For eight pairs of males, both males stayed in the corridor system for 48 h; for three pairs, both males left the corridor system before the 48 h were over; and for five pairs, one male completed the whole experiment while the other escaped.

The exploration score did not explain any of the movement variables in the corridor systems except the total time spent in the south patch, indicating a tendency between exploration score and corridor width. Less explorative voles tended to spend less time in the south patch when the corridor was wide (N = 23 data points, 370 ± 433 min, z = −1.7, *p* = 0.081), while more explorative voles spent more time in the south patch when the corridor was narrow (N = 20 data points, 598 ± 589 min) (Table 1). Furthermore, animals in the narrow corridor system entered the corridor faster (N = 16 voles, 6 ± 7 min, z = 5.6, *p* < 0.001, calculated using a ZANB model) than those in the wide corridor system (N = 17 voles, 35 ± 43 min) (Figure 2A), and voles in the wide corridor system spent more time in the corridor (N = 23 data points, 927 ± 388 min, t = 3.4, *p* = 0.003) than those in the narrow corridor system (N = 20 data points, 438 ± 319 min) (Table 1, Figure 2B,C).

## 4. Discussion

Contrary to what we hypothesized, the movement patterns of translocated common voles were not explained by the personality score measured in the behavioral experiments. The exploration scores were not found to predict any of the movement variables in the corridor systems. The total time spent in the south patch represents one exception, indicating that more explorative voles spent more time in the south patch when the corridor was narrow and less explorative voles spent less time in the south patch when the corridor was wide. This tendency was not confirmed by the results of other presented models. Landscape structure itself explained most of the observed variation in movement patterns, with voles moving faster through the narrow corridors.

### 4.1. Personality and Movement Behavior in Corridor Systems

As with previous studies involving this species, our personality tests also identified repeatable differences in the voles’ personality traits [55]. Additionally, our expectation that more explorative personality types will take greater risks and will be more likely to enter the narrow corridor more often or for longer periods of time compared to less explorative personality types was not met.

In a study on starling (*Sturnus vulgaris*) behavior, the speed of exploration did not correlate with movement, but the time spent on a perch did, indicating that the ability to predict movement depends on the behavioral trait investigated [10]. We identified one principal component and thus have only one behavioral trait. Other behavioral measurements may potentially be a better indicator for movement in large-scale, highly-structured habitats, as recent results by Schirmer et al. [12] indicate. Schirmer et al. [12] quantified boldness and exploration using an open field paradigm comparable to the one used here. However, they did not force the animals to enter the open field as we did. They found that the variables ‘latency to investigate an unknown, open area’ and ‘latency to emerge with the full body into an unknown, open area’ reflected boldness and that these variables affected home range, core area, and the overlap of both with conspecifics, while exploration of the open field (measured similarly to the present study) did not explain movement. However, more explorative bank voles (*Myodes glareolus*) moved shorter distances than less explorative voles, and bolder voles moved longer distances than shy voles. Neither behavioral traits correlated with each other, possibly due to the potential disconnect between boldness and exploration by the approach of behavior quantification in the field [12].

In accordance with many ecological studies using open field tests to measure activity and exploration [1], what we called an exploration score might instead measure passive or active coping mechanisms in a stressful situation. The open field test was originally developed to measure explorative versus emotional reactions towards an unknown area in small, nocturnal rodents [56]. Emotional responses may be linked to anxiety and are thus more likely to occur when animals are forcibly introduced into the arena rather than entering on their own [69]. Hence, the personality traits we measured may reflect how individuals cope under dangerous or stressful ecological conditions rather than their propensity to explore and use a corridor. In the corridor system, animals were free to choose the timing of their movement and could voluntarily decide to enter the corridor or stay in the relatively safe patch, hence this situation may have been perceived as less stressful.

About one third of the observed animals escaped from the corridor system grid after release. Neither the measured exploration score nor the distance from the trapping site or the corridor system could predict the escapees’ behavior. Exploration in the open field or the barrier tests may have different motivations than the choice of leaving a patch and entering a corridor or escaping the whole corridor system. Standardized test and large-scale movement observation may reflect two environments of different ecological risks, hence observed behavior may not be comparable. Furthermore, since the escape behavior was not affected by the corridor system, it might be a proxy for boldness (as opposed to exploration) in a large-scale environment. Since animals have to cope with their translocation to the corridor, we expected that animal personality, which is related to coping style, would explain movement behavior. However, we may have to refine our theory or question regarding how we measure personality traits. There are only a handful of studies evaluating the actual ecological relevance of standardized personality measurements, e.g., References [70,71,72]. Arvidsson et al. [70] compared the activity of great tits (*Parus major*) measured in a standardized testing environment to their exploration of a semi-natural enclosure, finding that the behavior measured in the standardized test was not correlated with that which was measured in the semi-natural environment and that they therefore did not measure the same behavior. Furthermore, Krebs et al. [72] found that in house mice (*Mus musculus*), some behaviors related to exploration measured in an open field test predicted the colonization of semi-natural enclosures, while behaviors more related to anxiety showed no such association. Lecorps et al. [71] also found that standardized tests for sociability were related to social proximity of house mice in home pens. Nonetheless, Schirmer et al. [12] found a relation between personality and home range size in stable populations of the closely related bank vole, accounting for two factors which were not fulfilled in our study: Behavioral quantification in the field and observations of stable unmanipulated populations.

The absence of an effect of exploration on movement in the corridor may also indicate that the tested animals do not represent the whole range of exploration phenotypes well, due to a trapping bias (for example). Passive trapping methods like baited traps may only motivate more explorative animals to enter a trap, hence individuals with very low exploration tendencies may be missing in our study [73].

The time voles spent in the south patch was affected by the month and the experimental day. Furthermore, we found a tendency of the month to affect the time animals stayed in the corridor. The effect of the experimental day may be related to different strategies for exploring an unknown habitat. Meanwhile, the motivation for reproduction among the males, as well as the resources for foraging [43,48] may have changed during the summer season, both of which could affect the vole’s movement behavior and motivation.

Taken together, our results showed that personality had no effect on corridor use. However, personality might nevertheless be relevant in motivating animal movement as is the case in dispersal processes. Connectivity will have an impact on the success of dispersal, especially since it depends inter alia on an animal’s capability to disperse. Departure, transience, and settlement are three consecutive dispersal stages [4]. By releasing all test animals as non-residents irrespective of their behavioral adaptation to dispersal, the trigger inducing departure was missing in the experimental setup and the observed movement may rather represent the transience stage. Moreover, our tested males may have been too old to exhibit dispersal behavior. Other studies investigated behavioral traits to form different functional groups of dispersers within populations [4,5,10,74,75,76], indicating that personality may affect an animal’s propensity to initiate movement processes such as dispersal.

### 4.2. Habitat Features Affecting Movement Behaviour

The movement behavior of voles differed in the two experimental setups investigated. Animals spent more time in the wide corridor than in the narrow one, which correlates positively with the number of completed trips between both patches, as well as the number of changes in direction while travelling through the corridor. Narrower corridors—and narrowness needs to be defined relative to the movement capabilities of the animal species—are more prone to prompt avoidance [77] or transient movement, such as dispersal, while wider corridors may be used as habitat patches for settlement, as well [34,35]. Corridor width determines whether corridors are used rather permanently as habitat or for transient movement [31]. Corridors may thus not only allow for movement, but also affect population dynamics [78] and predator-prey-interactions [79]. As a consequence, a corridor’s contribution to connectivity depends on the movement scale of its user, i.e., for small species, a corridor may represent a sink [17,80], whereas, for large species, it represents a structure allowing for dispersal. Moreover, space use can change for animals staying permanently in corridors, e.g., they can have smaller home ranges and higher territoriality than in broader habitats [80].

Andreassen et al. [35] studied the optimal width of corridors on root voles (*Microtus oeconomus*) and found that the intermediate corridor width (1 m) resulted in the highest connectivity and thereby challenged the general principle of ‘the-wider-the-better’ for corridor design (as discussed in Reference [35]). Since common voles in our study stayed longer in the wide corridor (3 m) than in the narrow one (1 m), the wide corridor may have been perceived as a suitable habitat for residential settlement. Wider corridors with the potential for settlement may thus have different functions, on a longer time scale, apart from connecting isolated patches, with generations of settled animals slowly drifting towards the other habitat and gene pools potentially mixing within the corridor. Transient movement, meanwhile, connects habitat patches within a fraction of the lifetime of the disperser. In Andreassen’s study [35] animals refused to enter the narrowest corridor (0.4 m), which possibly posed the highest predation risk. Animals travelling through corridors are repulsed by the borders, so an animal inside a wide corridor will reach the corridor’s borders less frequently and are therefore guided by the corridor’s geometry to a lesser extent than animals travelling in a narrow corridor. The movement through any elongated structure may be shaped by the repulsion of the edges, which consequently develops into an almost straight line, when the corridor is narrow (e.g., zig-zag-movement in Reference [35]).

In our study, animals in the narrow corridor system (1 m) entered the corridor faster than those in the wider corridor system. Corridors were entered earlier when patches and corridor were more dissimilar (10 m vs. 1 m) compared to wider corridors (10 m vs. 3 m). Due to the 1 m-wide extension, animals in the narrow system may have realized more quickly than those in the wide system that the patch is too small for settlement. Thus, animals in the wide system may have needed more time because a 3 m-wide extension may have been perceived as being part of the patch itself.

To conclude, our results confirmed the assumption that corridor characteristics affect the functionality of a corridor. Our corridor systems did not differ in vegetation height or density and had similar population densities before removal. This implies that both systems were of similar quality to be inhabited by common voles. Consequently, the corridors’ width influenced the movement behavior of male common voles. A width of about 1 m appears to be a critical threshold for common voles and other rodents [35] to enable connectivity of otherwise disconnected habitats or patches. Further studies should challenge other corridor characteristics, like vegetation density or characteristics of the matrix, to understand their influence on corridor use.

### 4.3. Implications for Conservation and Functional Connectivity of Corridors

Due to strong fragmentation of habitats through an increasing human population, the movement abilities of animals are increasingly important. The functionality of corridors, according to our results, depends on its characteristics and should thus be adjusted to the overall movement capabilities of a species. Palmer et al. [18] showed that connectivity depends on the variability in dispersal, suggesting that animal personality measures may have the potential to affect estimates of landscape connectivity. However, since we found no significant effect of the exploration score on the latency to enter the corridor, our results did not support the hypothesis that more explorative animals explore the corridors and both patches more quickly. Consequently, corridor design seems to affect the mobility of entire populations rather than certain personality types.

In summary, we found that landscape features such as corridor width affected the movement of common voles in corridors, but that animal personality did not. Thus, corridor geometries in relation to a species’ movement capabilities should be considered when planning corridors that should facilitate the dispersal of the target species.

## Figures and Tables

**Table 1:** Full models of movement observations and main effects. The animal ID and the paired male from the same corridor system were included as random effects. The following models were applied: Zero-altered negative binomial model (ZANB), a two-part model compiled with a hurdle count model; linear mixed effects model (LMM); and generalized linear mixed effects model (GLMM). Additionally, we analyzed the interaction of the exploration score (ES) and the corridor width (CW). Significant results and trends are displayed in bold. The marginal R^2^ includes fixed effects and the conditional R^2^ includes the full model with fixed and random effects.

Variable	Latency to Enter Corridor								Latency to Return to Start Patch after Visiting South Patch [min]				Total Time Spent in Corridor [min]				Total Time Spent in South Patch [min]			
Model, Assumed Distribution of Residuals	Hurdle, ZANB, N = 33 Animals								LMM, Gaussian, N = 21 Animals				LMM, Gaussian, N = 43 Data Points (N_Day 1_ = 22, N_Day 2_ = 21)				GLMM, Poisson, N = 43 Data Points (N_Day 1_ = 22, N_Day 2_ = 21)			
	Count Model Coefficients (Truncated Negative Binomial with Log Link)				Zero Hurdle Model Coefficients (Binomial with Logit Link)															
	Est.	SE	Z	*p*	Est.	SE	z	*p*	Est.	SE	t	*p*	Est.	SE	t	*p*	Est.	SE	z	*p*
**Intercept**	2.3	0.4	5.6	**<0.001**	−0.3	0.7	−0.5	0.651	588.3	552.4	1.1	0.320	102.7	168.9	0.6	0.547	6.7	1.0	6.6	**<0.001**
**Exploration Score**	0.03	0.2	0.1	0.882	0.3	0.4	0.7	0.470	152.5	230.4	0.7	0.518	26.0	69.8	0.4	0.713	−0.1	0.8	−0.2	0.854
**Corridor Width (wide)**	1.4	0.4	3.8	**<0.001**	1.2	0.8	1.5	0.139	617.3	560.4	1.1	0.300	464.6	136.2	3.4	**0.003**	−1.0	1.0	−0.9	0.348
Interaction ES*CW			removed				removed				removed				removed		−2.0	1.1	−1.7	**0.081**
**Month**	0.02	0.4	0.04	0.967	1.0	0.9	1.2	0.241	813.9	594.3	1.4	0.207	277.8	144.3	1.9	**0.070**	−2.6	1.2	−2.2	**0.027**
**Day**	not included								not included				110.9	70.3	1.6	0.130	−0.1	0.0	−6.7	**<0.001**
**Log(theta)**	0.39	0.3	1.2	0.236																
**marginal R^2^**	no random factors in ZANB included								0.18				0.40				0.34			
**conditional R^2^**	no random factors in ZANB included								0.53				0.74				1.00			

## Supplementary Materials

The following are available online at https://www.mdpi.com/2076-2615/9/6/291/s1: Figure S1: Experimental schedule; Figure S2: Movement tracks of all observed common voles; Table S1: Repeatability of four behavioral variables in the barrier test and the open field test; Table S2: Descriptive statistics of movement measurements of males in narrow and wide corridor systems; Table S3: Correlation matrix of all movement variables; and Table S4: Full models of movement observations and main effects.
