# Supplementary material for: Does Animal Personality Affect Movement in Habitat Corridors? Experiments with Common Voles (Microtus arvalis) Using Different Corridor Widths
Source: Animals (Basel). 2019 May 29;9(6):291. doi: 10.3390/ani9060291 (PMC6616401; doi:10.3390/ani9060291)
Supplement: Supplementary file 1 [file animals-09-00291-s001.pdf]

Supplement

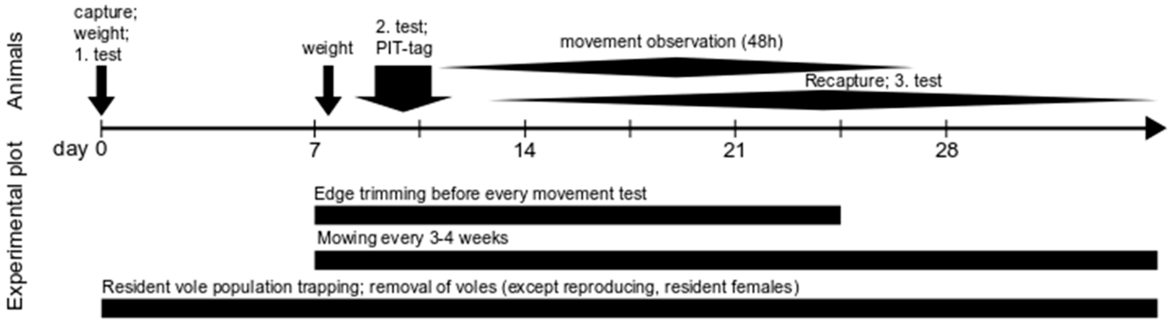

Figure S1: Experimental schedule: Overview of the experimental schedule including trapping, testing, and monitoring the test animals. Arrow width reflects the range of days when several steps were performed. For example, the width of the second test arrow indicates that males were tested between days 8-14.

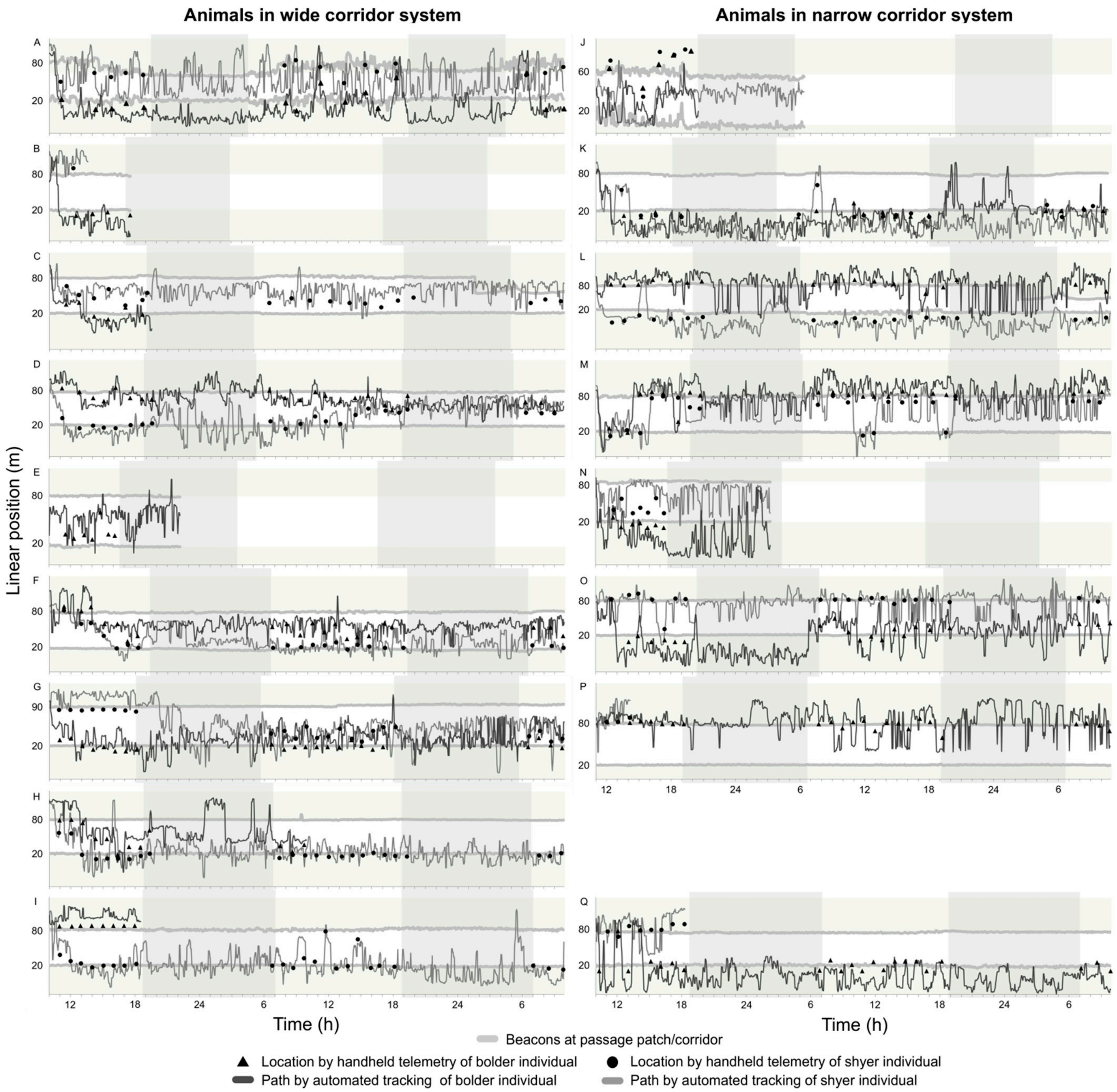

Figure S2: Movement tracks of all observed common voles. Movement tracks over time (48 h) in two experimental corridors connecting two habitat patches, with the starting patch above 80 m, the second patch below 20 m, and the corridor extending from 20 to 80 m. Vertical grey shading blocks represent nighttime. The locations of the voles were obtained every 3 min using automated telemetry and smoothed over 9 min by a running median (medium and dark grey lines). Locations were obtained from signal position along the axis parallel to the corridor. Beacons (VHF radio transmitters at known positions) at the actual passage point between patch and corridor mark a calculated boundary for recorded vole locations either in the patch or in the corridor (light grey lines at 20 m and 80 m). Actual vole locations recorded with handheld telemetry are represented by black circles and triangles. Each male pair consisted of a more explorative individual (black triangles and dark grey lines) and a less explorative individual (black circles and medium grey lines). In one experimental run, we had technical problems with the automated tracking. Thus, data collection stopped after 1820 hours (Supplement Figure 2E, N). During this run, one male in the wide corridor system was lost to predation (no tracking data, Supplement Figure 21E), one stayed within the narrow corridor system (Supplement Figure 2N), and the last two escaped from the corridor system after tracking was interrupted (Supplement Figure 2E, N).

Table S1. Repeatability (R) of four behavioral variables in the barrier test (B) and the open field test (OF). The repeatable variables are ‘crossing frequency’ (No. of crossings per min), ‘activity’ (1-0-sampling every 10 sec.) and ‘entering unsafe zone’ (yes/no latency). Shown are raw and conditional repeatability estimates with their corresponding confidence interval (CI). P-values were calculated by LRT (likelihood ratio test). Significant results are displayed in bold.

**Test**	**Behavioral variable**	**Raw R [CI]**	**p-value**	**Explanatory variables**	**t-value**	**p-value**	**Conditional R [CI]**	**p-value**
**B**	Crossing frequency	0.27	**<0.01**				0.35	**<0.001**
		[0.09, 0.45]		Season	-1.23	0.22	[0.17, 0.52]	
				Test interval	-1.23	0.22		
				Time of day	-0.77	0.44		
				Test-round	-0.74	0.13		
**B**	Activity	0.24	**<0.01**				0.31	**<0.001**
		[0.06, 0.43]		Season	-0.15	0.88	[0.11, 0.49]	
				Test interval	0.26	0.79		
				Time of day	-1.31	0.19		
				Test-round	-2.33	**0.02**		
**OF**	Entering unsafe zone	0.20	**0.02**				0.24	**0.02**
	(binary model)	[0, 0.40]		Season	-1.93	0.06	[0, 0.67]	
				Test interval	-1.53	0.13		
				Time of day	-0.82	0.41		
				Test-round	-0.26	0.80		
**OF**	Activity	0.32	**0.001**				0.39	**<0.001**
		[0.13, 0.50]		Season	0.70	0.49	[0.21, 0.57]	
				Test interval	-0.27	0.79		
				Time of day	-2.39	**0.02**		
				Test-round	-2.56	**0.01**		

The activity measurements from both the barrier and open field tests decreased with the test round. In the barrier test, activity was higher in the first test round (21.6 ± 6.1 incidences of activity) than in the second (20.2 ± 7.0 incidences of activity) and third rounds (16.2 ± 7.0 incidences of activity, LMM: $t = -2.3$, $p = 0.02$). Results were similar in the open field test; in the first round, voles were more active (17.5 ± 6.3 incidences of activity) than in the second (14.1 ± 7.7 incidences of activity) and the third round (11.6 ± 7.6 incidences of activity, LMM: $t = -2.6$, $p = 0.01$). Moreover, the activity in the open field test decreased throughout the day LMM: ($t = -2.4$, $p = 0.02$), and neither the season nor the test interval significantly influenced behavior.

Table S2. Descriptive statistics of movement and activity measurements of males in narrow and wide corridor systems (min: minutes, SD = standard deviation, Min = Minimum, Max = Maximum). All variables were collected from 22 voles from the first day and 21 voles from the second day. Exceptions: 'latency to enter the corridor for the first time' was measured for 33 voles, 'latency to arrive at the south patch for the first time' for 18 voles and 'latency to return to the start patch after visiting the south patch' was collected for 14 voles.

	**Wide Corridor System**				**Narrow Corridor System**			
	Mean	SD	Min	Max	Mean	SD	Min	Max
**Number of visits to the corridor**	20	12	1	38	21	12	3	39
**Total time spent in the corridor [min]**	927	388	69	1434	438	319	27	1140
**Number of visits to the north patch**	6	8	0	26	15	15	0	39
**Total time spent in the north patch [min]**	141	194	0	609	400	433	0	1083
**Number of visits to the south patch**	14	13	0	36	7	8	0	26
**Total time spent in the south patch [min]**	370	433	0	1335	598	589	0	1407
**Number of changes in movement direction while travelling**	130	56	11	216	56	41	5	150
**Latency to enter the corridor for the first time [min]**	35	43	0	156	6	7	0	21
**Latency to arrive at the south patch for the first time [min]**	221	230	57	783	153	221	21	669
**Latency to return to the start patch after visiting the south patch [min]**	1006	735	144	1914	649	651	105	1992
**Number of completed trips between both patches**	2	4	0	17	3	5	0	20

Table S3. Correlation matrix of all movement variables. Correlations were estimated with the function "rcorr" (package Hmisc, 2018). Rho values are on the top right side of the table (rho > 0.3 displayed in bold), respective p-values are on the bottom left side (significant p-values are displayed in bold).

	**Number of visits to the corridor**	**Total time spent in the corridor [min]**	**Number of visits to the north patch**	**Total time spent in the north patch [min]**	**Number of visits to the south patch**	**Total time spent in the south patch [min]**	**Number of changes in movement direction while travelling**	**Latency to enter the corridor for the first time [min]**	**Latency to arrive at the south patch for the first time [min]**	**Latency to return to the start patch after visiting the south patch [min]**	**Number of completed trips between both patches [day 1]**
**Number of visits to the corridor**		**0.44**	**0.46**	0.21	**0.58**	0.12	**0.49**	0.1	-0.14	-0.17	**0.35**
**Total time spent in the corridor [min]**	**0.010**		**0.36**	0.05	0.15	-0.19	**0.98**	0.12	0.2	-0.02	0.27
**Number of visits to the north patch**	**0.007**	**0.042**		**0.82**	-0.3	-0.53	**0.4**	0.23	**0.42**	-0.25	0.21
**Total time spent in the north patch [min]**	0.244	0.792	**<0.001**		-0.44	-0.56	0.09	**0.4**	**0.51**	-0.12	-0.01
**Number of visits to the south patch**	**<0.001**	0.399	0.093	**0.011**		**0.72**	0.17	-0.1	-0.57	-0.09	**0.43**
**Total time spent in the south patch [min]**	0.513	0.299	**0.001**	**0.001**	**<0.001**		-0.23	-0.13	-0.72	-0.01	**0.34**
**Number of changes in movement direction while travelling**	**0.004**	**<0.001**	**0.022**	0.620	0.333	0.197		0.15	0.26	0.02	0.21
**Latency to enter the corridor for the first time [min]**	0.593	0.508	0.194	**0.021**	0.592	0.471	0.415		0.12	-0.07	0.01
**Latency to arrive at the south patch for the first time [min]**	0.446	0.268	**0.015**	**0.003**	**0.001**	**<0.001**	0.151	0.490		0.43	-0.46
**Latency to return to the start patch after visiting the south patch [min]**	0.355	0.913	0.158	0.508	0.623	0.961	0.891	0.714	**0.012**		-0.78
**Number of completed trips between both patches [day 1]**	**0.044**	0.130	0.232	0.973	**0.013**	**0.050**	0.247	0.947	**0.008**	**<0.001**	

Table S4. Full models of movement observations and main effects. The animal ID and the paired male from the same corridor system were included as random effects. The following models were applied: linear mixed effects model (LMM) and generalized linear mixed effects model (GLMM). Additionally, we tested the interaction of exploration score (ES) and corridor width (CW). Significant results are displayed in bold. Marginal R^2 includes fixed effects, conditional R^2 includes the full model with fixed and random effects.

The models of 'latency to arrive at the south patch for the first time' [min] and 'number of completed trips between both patches' were difficult to interpret due to the distribution of the data, and neither transformation nor model fitting made their interpretation easier. Due to strong correlations (rho > 0.8), the number of changes in direction while travelling (strong correlation with the total time spent in the corridor [min]) and the number of visits to the north patch (strong correlation with the total time spent in the north patch [min]) were not modelled.

**Variable**	**Total time spent in the north patch [min]**				**Number of visits to the corridor**			
**Model, assumed distribution of residuals**	GLMM, poisson, N=43 data points				LMM, gaussian, N=43 data points			
	Est.	SE	Z	p	Est.	SE	t	p
**Intercept**	5.0	0.7	7.3	**<0.001**	12.4	6.0	2.1	**0.045**
**Exploration Score**	0.1	0.4	0.4	0.691	-1.1	2.5	-0.5	0.648
**Corridor Width (wide)**	-0.4	0.7	-0.6	0.533	-2.3	4.8	-0.5	0.636
**Interaction ES*CW**				removed				removed
**Month**	0.3	0.7	0.4	0.717	6.8	5.1	1.3	0.197
**Day**	-0.3	0.0	-13.6	**<0.001**	2.9	2.5	1.2	0.258
**Marginal R^2**	0.04				0.09			
**Conditional R^2**	1.00				0.59			

**Variable**	**Total time spent in the south patch [min]**				**Number of visits to the south patch**			
**Model, assumed distribution of residuals**	GLMM, poisson, N=43 data points				GLMM, poisson, N=43 data points			
	Est.	SE	Z	p	Est.	SE	z	p
**Intercept**	6.4	1.1	6.0	**<0.001**	1.2	0.6	2.2	**0.028**
**Exploration Score**	-1.2	0.6	-2.0	**0.042**	-0.6	0.3	-1.9	**0.052**
**Corridor Width (wide)**	-0.9	1.1	-0.8	0.431	0.4	0.6	0.6	0.542
**Interaction ES*CW**				removed				removed
**Month**	-1.9	1.2	-1.6	0.102	-0.4	0.6	-0.6	0.554
**Day**	-0.1	0.0	-6.7	**<0.001**	0.3	0.1	3.3	**<0.001**
**Marginal R2**	0.24				0.19			
**Conditional R2**	1.00				0.92			
